# Origins and Importance of Intragranular Cracking in Layered Lithium Transition Metal Oxide Cathodes

**DOI:** 10.1021/acsaem.4c00279

**Published:** 2024-04-25

**Authors:** Jędrzej K. Morzy, Wesley M. Dose, Per Erik Vullum, May Ching Lai, Amoghavarsha Mahadevegowda, Michael F. L. De Volder, Caterina Ducati

**Affiliations:** † Department of Materials Science and Metallurgy, University of Cambridge, 27 Charles Babbage Road, Cambridge CB3 0FS, United Kingdom; ‡ Institute for Manufacturing, Department of Engineering, University of Cambridge, 17 Charles Babbage Road, Cambridge CB3 0FS, United Kingdom; § Faraday Institution, Quad One, Harwell Science and Innovation Campus, Didcot OX11 0RA, United Kingdom; ∥ Department of Physics, Norwegian University of Science and Technology, Ho̷gskoleringen 1, Trondheim 7034, Norway; ⊥ Sintef Industry, Trondheim 7034, Norway

**Keywords:** electrochemistry, Li-ion batteries, cathodes, electron microscopy, electron energy loss spectroscopy

## Abstract

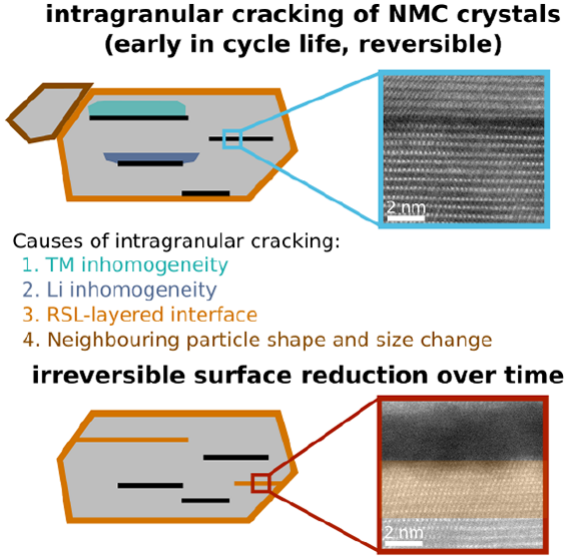

Li-ion batteries have a pivotal role in the transition toward electric transportation. Ni-rich layered transition metal oxide (LTMO) cathode materials promise high specific capacity and lower cost but exhibit faster degradation compared with lower Ni alternatives. Here, we employ high-resolution electron microscopy and spectroscopy techniques to investigate the nanoscale origins and impact on performance of intragranular cracking (within primary crystals) in Ni-rich LTMOs. We find that intragranular cracking is widespread in charged specimens early in cycle life but uncommon in discharged samples even after cycling. The distribution of intragranular cracking is highly inhomogeneous. We conclude that intragranular cracking is caused by local stresses that can have several independent sources: neighboring particle anisotropic expansion/contraction, Li- and TM-inhomogeneities at the primary and secondary particle levels, and interfacing of electrochemically active and inactive phases. Our results suggest that intragranular cracks can manifest at different points of life of the cathode and can potentially lead to capacity fade and impedance rise of LTMO cathodes through plane gliding and particle detachment that lead to exposure of additional surfaces to the electrolyte and loss of electrical contact.

## 1 Introduction

The wider adoption of electric transportation relies on the development of Li-ion batteries (LIBs) with higher energy density, faster charging rates, and better cost efficiency. Currently, the performance of cathode active materials (CAMs) is a limiting factor in the energy density, longevity, and cost efficiency of LIBs. Ni-rich layered transition metal oxides such as NMCs (LiNi_*x*_Mn_*y*_Co_*z*_O_2_, where *x* + *y* + *z* = 1) are promising CAM candidates owing to their large specific capacity and lower cost (due to lower Co content) compared to lower Ni counterparts.^1,2^ Such Ni-rich NMCs are considered attractive cathode materials for electric vehicles by major manufacturers, such as Northvolt,^3^ SK Innovation, and LG Chem.^4^ However, deterioration of the performance in NMCs is known to be accelerated with increasing Ni content and increasing cutoff voltage.^5,6^ The underlying mechanisms of this degradation have continued to be extensively studied. Briefly, at a high state of charge (SOC), reactive oxygen is released at NMC surfaces, which results in an irreversible structural transformation of the initial layered (*R*3-*m*) structure into an oxygen-deficient rock-salt-like layer (RSL), which is accompanied by a reduction in transition metal (TM) oxidation states at the surface. The RSL formation is associated with a loss of the active material mass (RSL is electrochemically inactive) and an increase in charge-transfer impedance of the cathode.^7−11^ The released reactive oxygen chemically oxidizes electrolyte components, which in turn may lead to the formation of additional resistive layers on the cathode (cathode–electrolyte interphase) and/or the formation of acid species in the electrolyte (including HF).^12−15^ Resulting acid species are then thought to be a cause of TM dissolution from the cathode.^16,17^ The dissolved TMs can catalyze deterioration and further growth of the anode solid–electrolyte interphase (SEI) contributing to loss of active Li inventory causing capacity fade.^17−20^ While most of capacity loss in the early battery life is attributed to active Li inventory loss, later in cycle life it is the cathode degradation that contributes to performance loss due to cathode capacity fade and impedance rise.^7,18^ This degradation can be caused by the abovementioned RSL evolution, but also cracking, which is thought to lead to isolation of particles and aggravation of other abovementioned degradation mechanisms due to exposure of fresh surfaces for oxygen release, TM dissolution, RSL formation, and side reactions with the electrolyte.^21−24^ The cracking is often attributed to anisotropic lattice breathing:^6,21,22,25^ during delithiation, the *a* lattice parameter monotonically shrinks, whereas the *c* lattice parameter initially increases and then, at high SOC, drastically decreases (collapses).^26−28^ Within cracking, two types are distinguished: intergranular cracking (between primary crystals), which is commonly discussed,^29−35^ and intragranular cracks (within primary crystals) for which the formation mechanisms and importance are much more elusive.

It has been suggested that the intragranular cracks are caused by local stress at interfaces between an electrochemically inactive rock-salt phase and active (expanding and contracting) layered phases within NMC crystals, caused by a dynamic lattice mismatch.^36,37^ Such stress would be highest at high SOC (biggest mismatch in lattice parameters of up to 3.5% at 80% SOC)^38^ where the particles would irreversibly fracture (with the rock-salt phase lining the crack) releasing this stress.^36,37^ A different mechanism proposed in the literature is based on intrinsic properties of the layered Ni-rich NMC: edge dislocations and other defects cause local stress, which combined with lowered structural stability and mechanical strength of NMC at high SOC causes intragranular cracking (or initially just an increase in the (003) layer spacing).^39,40^ In the literature, the intragranular cracks are typically studied in polycrystalline NMCs after cycling (e.g., 100 cycles or more) in the charged state.^36,40^

This work comprises a comprehensive electron-microscopy-based study into the nature of intragranular cracks in NMC cathodes. Both polycrystalline (PC) and single-crystal (SC) NMCs are investigated to elucidate the effect of particle morphology on cracking. Samples in early life (after formation cycles) as well as after extended cycling (300 cycles) are analyzed. The influence of stresses involved during calendering on intragranular cracking is also probed. Through the use of various electron microscopy imaging and spectroscopy techniques, we provide novel insights into the mechanisms of intragranular crack formation and their importance for the NMC cathode performance. We find that intragranular cracks do not have a single cause but rather arise from stress induced by a plethora of inhomogeneities within NMC particles (primary and secondary). Furthermore, intragranular cracks form early in cycle life, are initially reversible, and do not immediately contribute to performance fading; however, we identify evidence of material degradation initiated by intragranular cracking that likely contributes to performance loss during extended cycling.

## 2 Results

### 2.1 Intragranular Cracking Takes Place in Early Cycle Life

**Figure 1:**
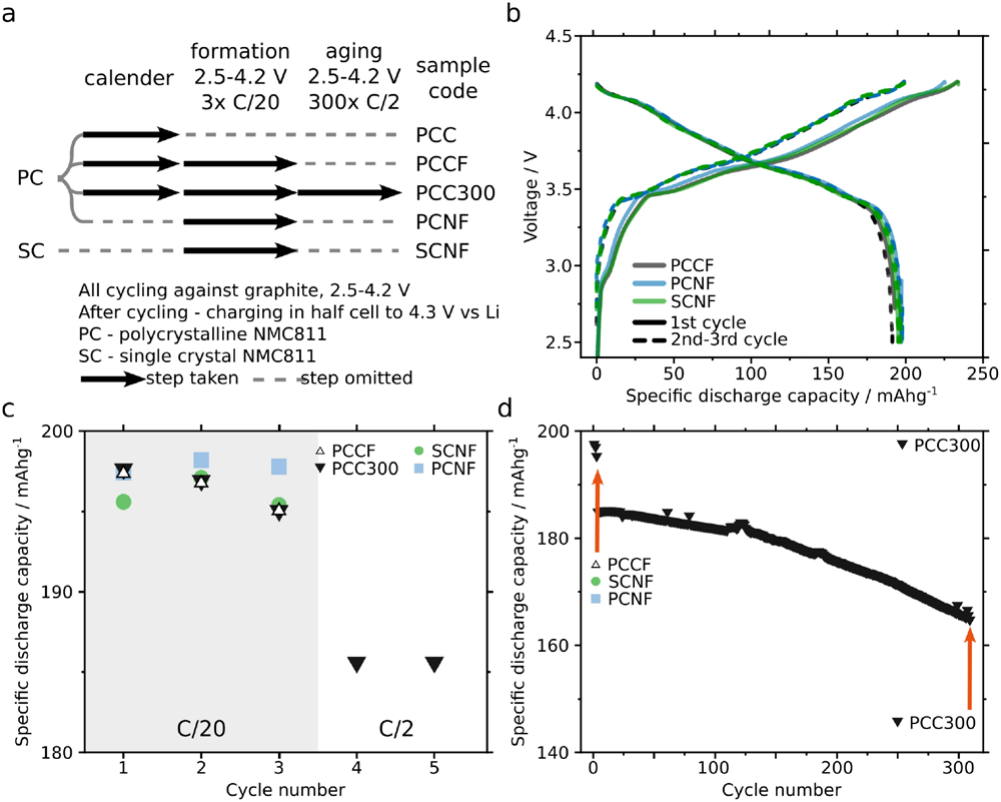
Sample set details and electrochemistry. (a) Schematic highlighting the differences between specimens and conditions applied. (b) Voltage profiles for the first three cycles for PCCF, PCNF, and SCNF. PCC300 is not shown as the protocol, and results are the same as for PCCF. (c,d) Specific discharge capacity in the early cycles (c) highlighting similar behavior regardless of specimen type (morphology and calendering) and during the 300 cycles for PCC300 (d). In (d), two red arrows show graphically the number of cycles with which each specimen was aged with. Cycles 1–3 are conducted at C/20 rate, 3–303 at C/2, and 2.5–4.2 V vs graphite. Specific discharge capacity of PCC300 decays steadily, losing 20.2 mAh/g compared to the first C/2 cycle (∼0.037%/cycle, 11% total), which is similar to our previous results (5.5%, over 150 cycles, ∼ 0.037%/cycle).^7^

**Figure 2:**
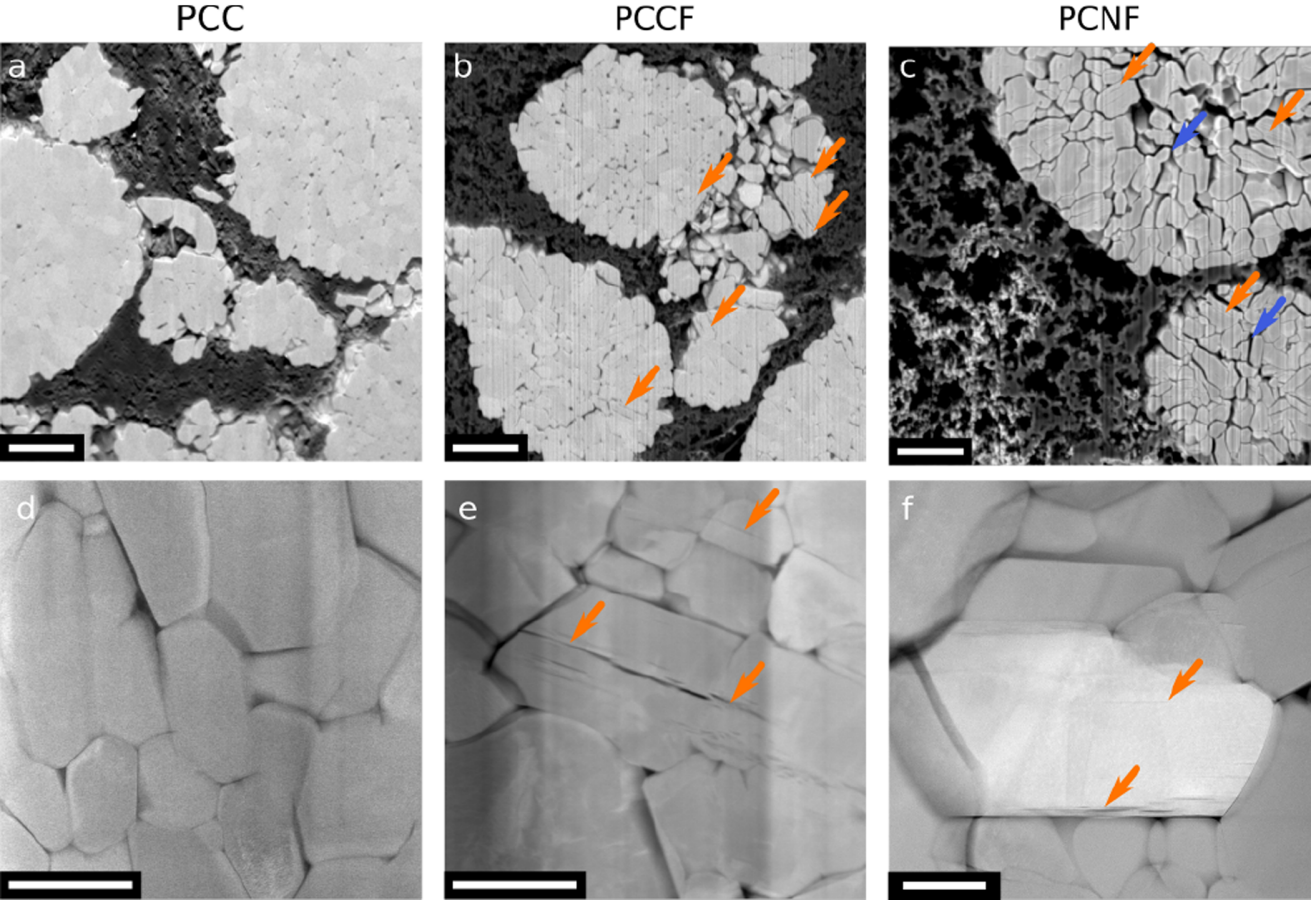
Representative SEM and HAADF-STEM images of the calendered pristine electrode (PCC; a,d); calendered, formed, and charged to 4.3 V (PCCF; b,e); noncalendered formed and charged to 4.3 V (PCNF; c,f) polycrystalline NMC811 samples. Orange arrows indicate intragranular cracks. Blue arrows in (c) highlight intergranular cracking that is affecting the grain boundaries within secondary particles more severely than for calendered electrodes (a,b). (a–c) FIB-SEM cross-sectional image taken with a backscattered electron detector and (d–f) HAADF-STEM images. Vertical lines in (a–c,e) are curtaining artifacts from the FIB milling process (also faintly visible at other angles in d and f). Scale bars: (a–c) 2 μm, (d–f) 500 nm.

A comprehensive sample set was prepared based on commercial NMC811 (LiNi_0.8_Co_0.1_Mn_0.1_O_2_; see Section 4 for more details) with rationally designed cycling conditions chosen to avoid exaggerated degradation, e.g., by high temperature or high upper cutoff voltage. Figure 1a shows the sample set and sample codes used throughout this work as well as the measured typical NMC811-graphite cell behavior (Figure 1b–d). Most of the specimens are investigated in the charged state (4.3 V vs Li; see Section 4) and consist of polycrystalline NMC811 unless otherwise specified. The full cells exhibit electrochemical behavior expected for NMC811 cells. In this work, we compare samples after just 3 slow formation cycles as well as after extended cycling (300 cycles). Noncycled, calendered electrode (PCC) and formed electrodes (PCNF – noncalendered and PCCF – calendered) were imaged using a high-angle annular dark-field scanning transmission electron microscope (HAADF-STEM) and a scanning electron microscope (SEM). Representative micrographs are shown in Figure 2. After calendering, but before cycling (PCC), particles show no signs of intragranular cracking (Figure 2a–d), and some signs of intergranular cracking which is a result of calendering.^7,34,41^ In contrast, just after three formation cycles, in the charged state, intragranular cracks in NMC811 are evident in cross-sectional SEM (Figure 2b,c) and STEM imaging (Figure 2e,f), regardless of whether the specimen was calendered (PCCF, Figure 2b,e) or not (PCNF, Figure 2c,f) and regardless of sample morphology (PCCF and PCCNF in Figure 2 and SCNF in Figure S1). This challenges notions in the literature that intragranular cracking is a clear sign and significant cause of degradation.^36,40,42^ After three slow formation cycles, intragranular cracks are common, but the battery has not yet undergone significant performance decay (see Figure 1 and our previous work).^7^ Samples in the discharged state (after aging) rarely show intragranular cracking (see Figures S1 and S2), suggesting high levels of reversibility, where intragranular cracks form upon charge (with shrinkage of unit cell volume for NMC crystals) and seal during discharge (when the crystals expand again). It is important to note that by sealing of intragranular cracks we mean bringing back the two faces of an intragranular crack close enough together that they are indistinguishable from the unaffected material. However, the study of the atomistic nature of the interface between the two faces of a sealed crack is outside the scope of this publication. Single-crystalline, noncalendered NMC811 also exhibits clear signs of intragranular cracking after formation, in the charged state but not in the discharged state, as shown in Figure S1. Local stress arising from anisotropic expansion and contraction of neighboring primary particles in polycrystalline secondary particles is therefore not necessary for the formation of intragranular cracks. It is important to note that intragranular cracks are always observed to be parallel to the (003) planes of the layered NMC structure,^36,37,40^ so intragranular cracks can only be seen in micrographs if the crystals are imaged along the (003) planes. Therefore, as the orientation of crystals in the samples is random, electron microscopy underestimates the amount of intragranular cracking. This is in particular true for SEM imaging, which cannot resolve the smallest cracks (which can be <2 nm in thickness, see further). Interestingly, as shown in Figure 2c, the uncalendered secondary PCNF particles show grain boundaries more severely affected by intergranular cracking compared to calendared PCC or PCCF electrodes. A discussion on this can be found in Note S1, but briefly, this may be an effect of the carbon–binder matrix being compressed during calendering and the reason for better capacity retention and less impedance rise of calendered electrodes reported in the literature.^41^

### 2.2 Open and Closed Intragranular Cracks

**Figure 3:**
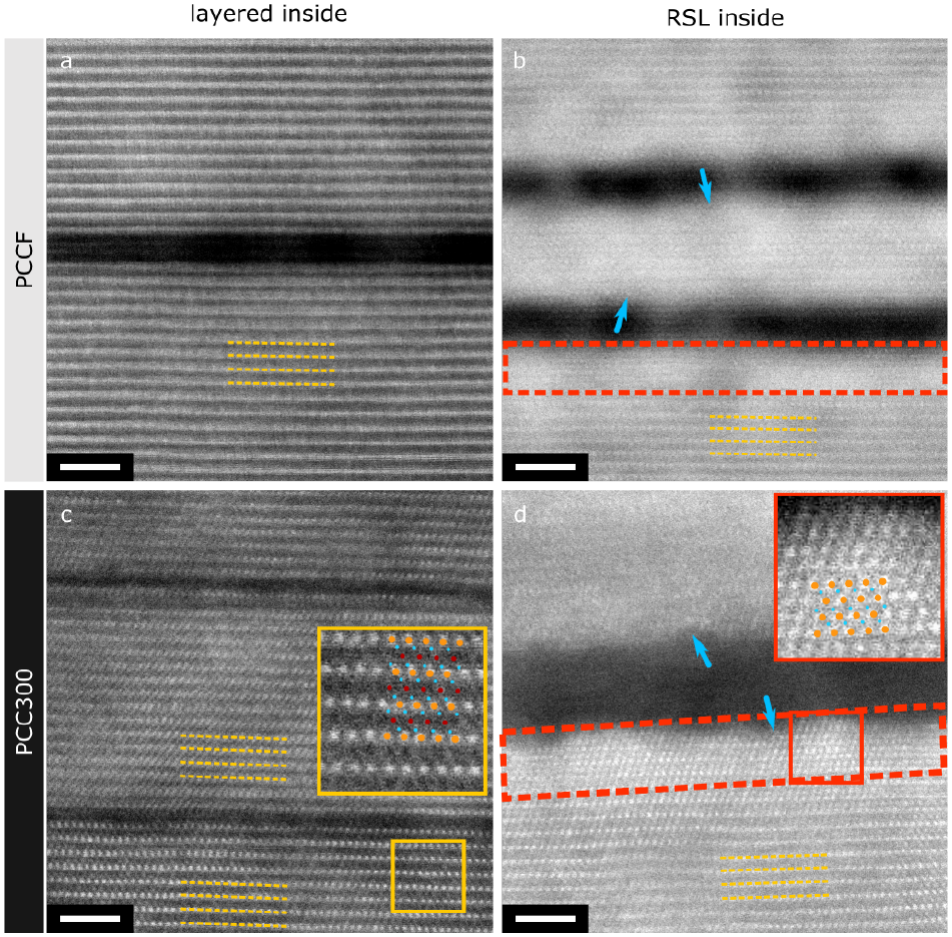
HAADF-STEM images of atomic structure of intragranular cracks in NMC811 samples: (a,b) PCCF (formed), (c,d) PCC300 (cycled). Left column shows representative examples of intragranular cracks with a layered structure at their inner surfaces, while the right column shows examples of cracks in which the inner surface has transformed into an RSL. All images are shown at the same magnification. Scale bar: 2 nm. Yellow dashed lines highlight the layered structure in the vicinity of intragranular cracks. Red dashed rectangles highlight the reconstructed RSL at the inner surfaces of intragranular cracks. Insets in (c) and (d) show magnified views of the layered (*R*3̅*m*, view along the [100]_layered_ axis) and rock-salt (*Fm*3̅*m*, view along [−110]_rock-salt_) structures, respectively. Orange circles – TM, teal – O, and red – Li positions. Blue arrows point toward irregular inner surfaces of cracks that have undergone transformation to RSL.

Figure 3 shows high-resolution HAADF-STEM micrograph examples of intragranular cracks in samples after formation (PCCF) and aging (PCC300). At the atomic scale, the intragranular cracks either appear as thin openings of the layered lattice along the (003) planes with widths on the order of a few *d*_003_ spacings (*d*_003_ = 0.47 nm), i.e., 0.6–2 nm (Figure 3a,c), or as wider, RSL-lined gaps with widths ranging between 1.5 and 3 nm (Figure 3b,d). In both formed and cycled specimens in the charged state (as exemplified by PCCF and PCC300 in Figure 3), an RSL is observed on the inner surfaces of some, but not all, intragranular cracks. This directly contradicts previous hypotheses from the literature that ascribed intragranular cracking directly and primarily to interactions of the rock-salt and layered phases.^36,37^ The results shown here indicate that intragranular cracks can exist, even after 300 cycles, without their inner surfaces irreversibly transforming into the rock-salt phase. The lack of RSL inside some of the intragranular cracks suggests that the inner surfaces of these particular intragranular cracks were not directly in contact with the electrolyte, as it is generally accepted that the layered-RS transformation depends on the crystal planes and the electrochemical cycling and is highly accelerated by the presence of electrolyte at the interface. The detailed discussion about the role of the electrolyte in the layered-RS transformation is beyond the scope of this study and is explored in the literature.^7,11,43,44^ In a case where an intragranular crack is “closed,” i.e., it is fully contained within a crystal and is not flooded with electrolyte, its inner surfaces will remain layered. Other cracks that are “open” and can be filled with electrolyte will be able to release oxygen, resulting in irreversible surface reduction and structural transformation. This can occur already in the first few (formation) cycles, as shown in Figure 3b. The distinction between “open” and “closed” intragranular cracks is significant, as it therefore implies that RSL on the surfaces of “open” intragranular cracks will be retained upon discharging (and further cycling), with an irreversible loss of the active layered phase. On the other hand, if the “closed,” nontransformed cracks fail to seal perfectly upon discharge, it could result in locally increased lattice spacing. Both these mechanisms could explain the observation of “premature cracks” shown in the work of Lin et al.,^36^ where the premature cracks are either regions of increased lattice spacing of the layered phase, or a line of rock-salt phase confined within a crystal. Intriguingly, intragranular cracks are expected to initially have smooth, straight inner surfaces as they are simple openings of the crystal lattice along the (003) planes. However, “open” cracks lined with the RSL typically have rough inner surfaces, with variations to apparent RSL thickness across the interface (Figure 3b,d, blue arrows). This observation could be either a result of TM dissolution or of how the layered-RSL transformation occurs, wherein atoms (especially TMs) of the original layered structure have to rearrange themselves to form the RSL, both leading to a change in surface topology, which has not been previously explored in the literature.

### 2.3 Local Microstructure and Composition Affect Intragranular Cracking

**Figure 4:**
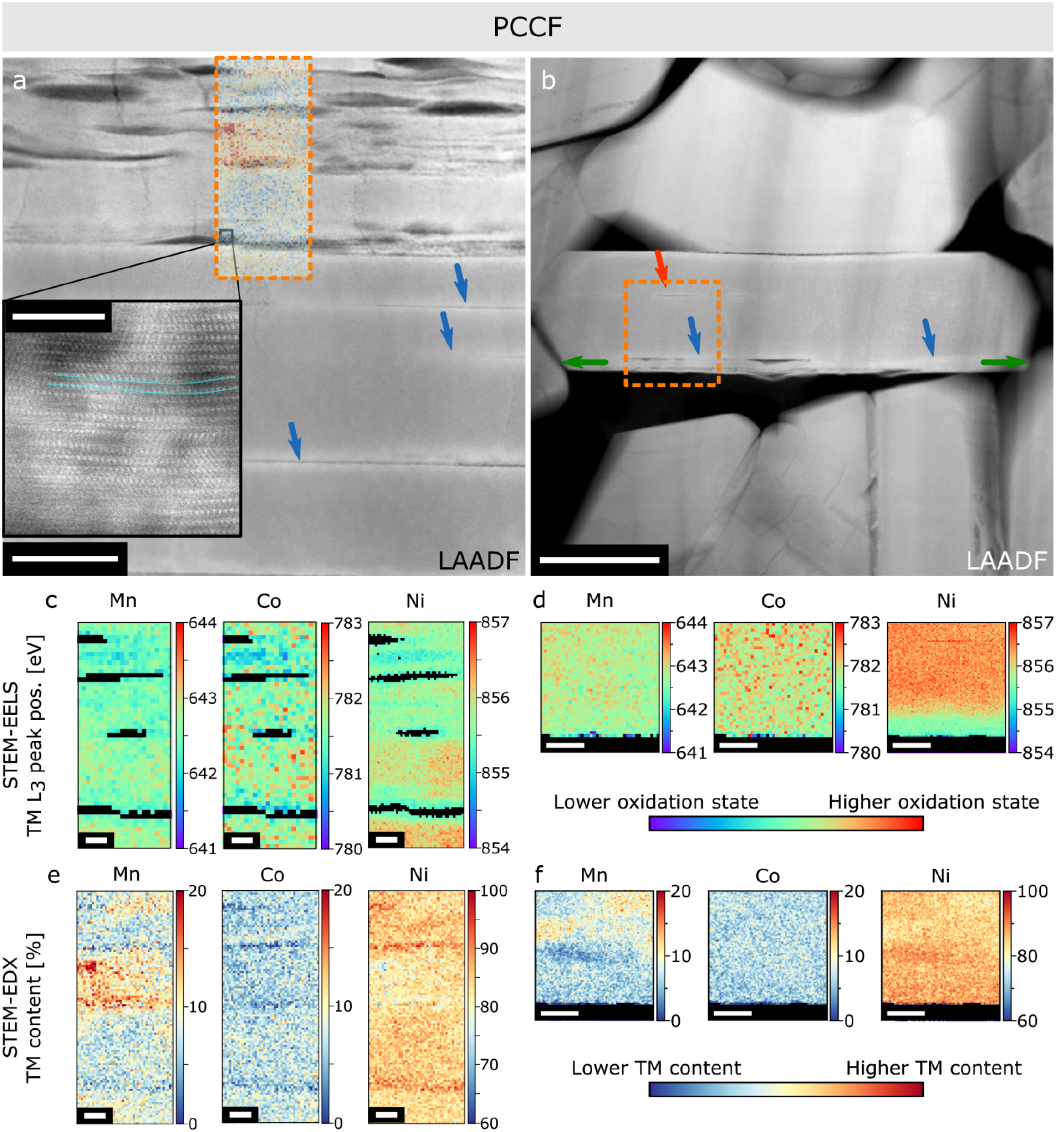
(a,b) LAADF-STEM images and (c,d) EELS and (e,f) EDX maps of two particles from the same secondary particle in the PCCF sample (see Figure S3). The (003) planes are oriented close to horizontal in both images. Blue arrows in (a,b) highlight strained (brighter) regions. Inset in (a) highlights lattice distortions around intragranular cracks. (c,d) EELS maps of TM L_3_ peak position for Mn, Co, and Ni for regions highlighted with orange rectangles in (a) and (b), respectively. (e,f) EDX maps of TM distribution for Mn, Co, and Ni for the same regions as (c,d). The Mn map from (e) is used to color the orange rectangle in (a) to highlight the correlation between intragranular cracks and TM inhomogeneity. Scale bar in (a) 50 nm, (b) 200 nm, inset in (a) 5 nm, (c,e) 10 nm, and (d,f) 50 nm. HAADF-STEM images of the same areas as parts (a,b) are shown in Figure S4 for comparison. Effects of FIB curtaining are seen in (b) at about 10° angle from vertical.

Thus far, we have shown that intragranular cracks are formed after just three formation cycles, irrespective of particle morphology (PC vs SC) and electrode compression (calendered vs noncalendered), and can be found both with and without RSL on their surfaces. These findings suggest that the cause of intragranular cracking is more complicated than originally proposed in the literature. To provide more insight into the origins of intragranular cracks, we investigated examples of NMC crystals from PCCF and PCC300 samples using ADF-STEM imaging as well as electron energy loss spectroscopy (EELS) and energy-dispersive X-ray spectroscopy (EDX) mapping. Lower magnification images of the TEM lamellae used as well as the regions where they were extracted from are shown in Figure S3. Figure 4 shows low-angle annular dark-field STEM (LAADF-STEM) images as well as EELS and EDX maps of two crystals within the same secondary particle (Figure S3). Briefly, LAADF-STEM images contain additional contrast from diffraction effects, including strain, compared with traditionally employed high-angle equivalent (HAADF-STEM) images. Therefore, it is possible to simultaneously map intragranular cracking and strain in the NMC. All LAADF images have HAADF counterparts (where contrast is proportional to thickness and atomic number) of the same areas shown in Figures S4 and S5 for comparison. The (003) planes of NMC in all panels in Figure 4 are oriented close to the horizontal. Figure 4a shows an inhomogeneously cracked particle with a heavily damaged top part (multiple delaminations, lattice distortions, bright strain contrast near intragranular cracks) and a less affected bottom part with thinner intragranular cracks. EELS L_3_ peak position maps of the TMs (Figure 4c,d) show a reduction of Ni around the cracks (lower L_3_ peak positions, see Section 4 for more details) indicating that most of the intragranular cracks in this particle were accessible to electrolyte and had their inner surfaces transformed into RSL over the three formation cycles.^45−47^ The maps of Co and Mn EELS L_3_ peak positions do not show significant chemical shifts and, hence, no significant reduction. This is expected as Ni is reduced more easily in NMC811.^7^ EDX maps (Figure 4e,f) show an inhomogeneous TM distribution, which is likely to be an effect of imperfect synthesis and has been observed in pristine particles in this commercial material (Figure S6). Given that NMCs are typically made by similar synthetic methods (i.e., coprecipitation), this observation is relevant to most commercial NMCs. Bands (parallel to the (003) planes) of compositions close to NMC811 dominate across the sample but some bands with higher Mn content are present in-between cracks. We propose this Mn–Ni inhomogeneity to be one of the causes of local stress leading to intragranular cracking. Lower Ni content NMCs exhibit smaller lattice contraction and expansion amplitudes in a charge–discharge cycle for a set upper cutoff voltage.^23,48,49^ At high SOC, two regions of different compositions would exhibit stress and strain at the interface, which may be released in the form of intragranular cracks at that interface. Notably, as will be discussed later, TM inhomogeneity is not strictly necessary for intragranular cracks to form and could simply be another contribution that induces their formation. Alternatively, TM inhomogeneity as a source of additional strain/stress could simply determine where the intragranular cracks would happen upon a larger strain/stress from other sources. The higher apparent Ni content inside of the intragranular cracks is noted in Figure 4e. This is a data processing artifact due to low signal-to-noise ratio in the mostly empty crack regions. This leads to noisy data, which combined with ∼8 times more Ni than Co or Mn, leading to overestimation of the Ni content.

Figure 4b,d,f shows a different primary crystal within the same secondary particle as the one in Figure 4a,c,e (see Figure S3). The crystal shows much less severe intragranular cracking (highlighting inhomogeneity of cracking within a secondary particle), confined to a region near the bottom edge of the crystal and a minor crack deeper in the crystal (red arrow in Figure 4b). Again, a bright strain contrast is seen in the vicinity of cracks (Figure 4b). EELS maps (Figure 4d) show Ni reduction of an ∼30 nm thick band near the bottom edge of the crystal. The reduced band is too thick to be solely due to outer surface layer reduction, as on the (003) terminated facets the RSL is typically 2–3 nm thick for these cycling conditions (see Figure S7). Rather, this feature is due to the transformation of the outer surfaces as well as the transformation of the inner surfaces of intragranular cracks close to that edge. Co and Mn maps show a similar trend as in Figure 4c. Conversely, an intragranular crack deeper in the primary crystal (red arrow in Figure 4b) does not exhibit any TM reduction, indicating that it is “closed” (Figure 4d). The “closed” crack is likely caused by TM distribution inhomogeneity as shown in Figure 4f. However, TM inhomogeneity is not present near the bottom edge of this particle and therefore cannot be the source of strain responsible for cracking in that region. An alternative explanation can be based on the local neighborhood of the crystal in question. Strain is seen as a band extending across the whole crystal in the LAADF image (Figure 4b, blue arrows), which may be a result of local stress coming from anisotropic expansion and contraction of this primary particle and its neighbors that are directly in contact. In this way, there is additional stress caused by the confinement of the bottom band of the particle and the expansion/contraction of the neighbors leading to preferential cracking in that region. It is worth noting that not all of the strained region is cracked, suggesting that strain is likely a cause, not an effect, of cracking.

**Figure 5:**
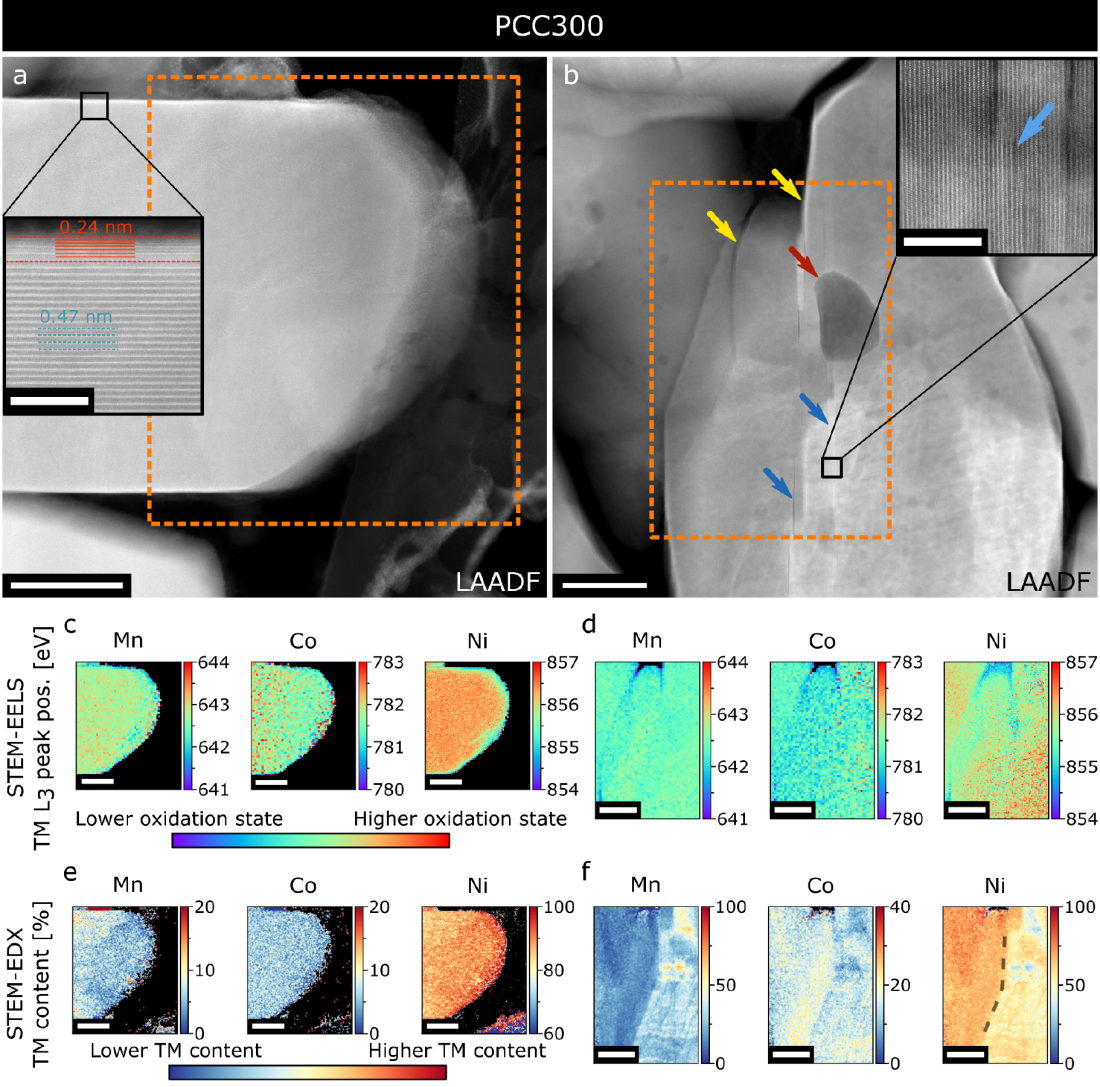
(a,b) LAADF-STEM images of two crystals from the PCC300 sample. Orange rectangles highlight areas where EDX and EELS mapping were performed. Blue arrows in (b) highlight intragranular cracks, and red arrow points toward an intragranular pore, and yellow arrows to steplike features on the particle surface. (c,d) EELS TM L_3_ peak position maps for Mn, Co, and Ni from the regions highlighted in (a) and (b), respectively. (e,f) EDX maps of TM distribution for Mn, Co, and Ni from the same regions. Scale bar in (a–f) 50 nm, insets in (a,b): 5 nm. (003) planes are close to horizontal in (a,c,e) and vertical in (b,d,f). HAADF-STEM images of the same areas as (a,b) are shown in Figure S5.

**Figure 6:**
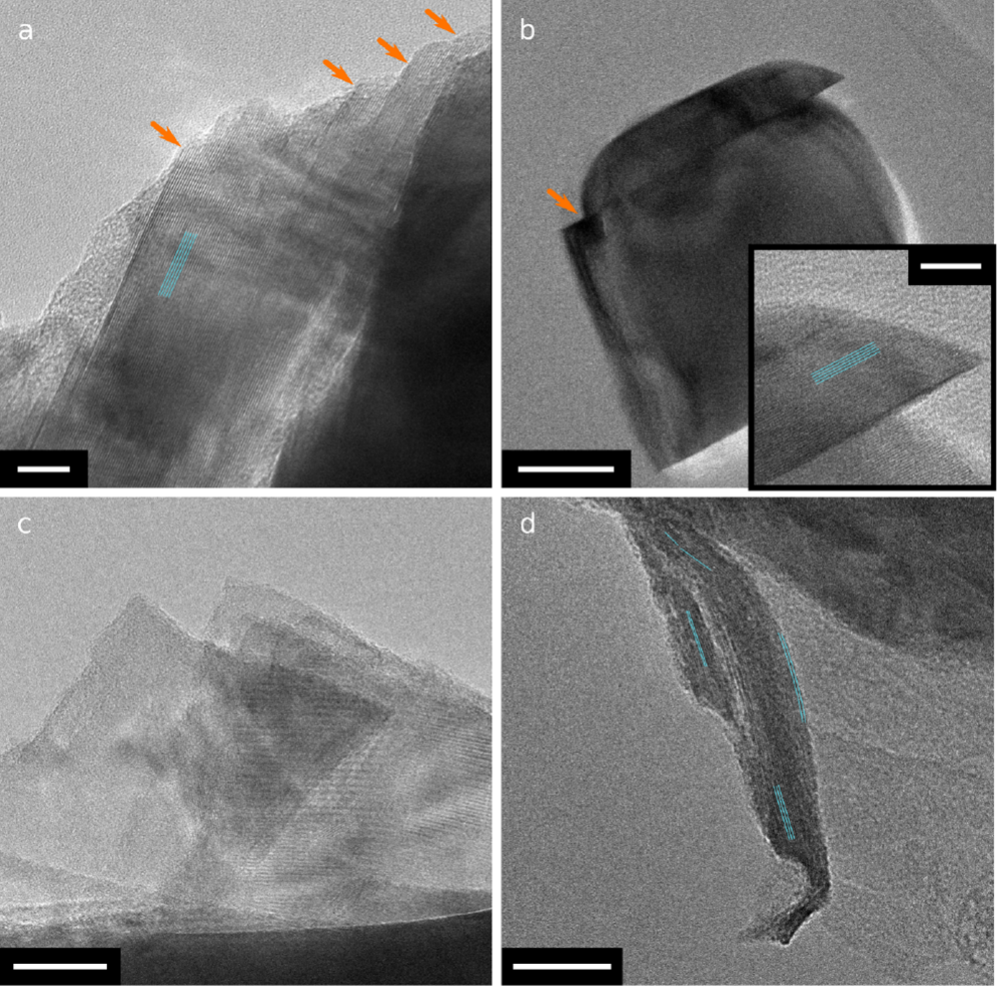
Bright-field TEM images of NMC811 samples after various electrochemical aging protocols were performed in the discharged state. Teal lines highlight the direction of (003) planes, if visible. (a) Plane gliding in NMC811 after 100 cycles against graphite 2.5–4.2 V, orange arrows highlight steplike features where (003) planes have glided. (b,c) Gliding of part of a crystal (b) and formation of flakes (c) after 100 cycles against graphite 2.5–4.3 V. (d) Delamination and deformation of part of a crystal along (003) planes after 600 cycles against graphite 2.5–4.3 V. Scale bars: (a) 10 nm; (b) 50 nm, inset: 10 nm; (c, d) 20 nm.

Figure 5 shows similar data for an aged specimen (PCC300). An inset in Figure 5a shows a high-quality layered structure within the particle, with a thin (∼1.5 nm) RSL on the surfaces parallel to (003) planes as well as a slightly thicker (∼3 nm) RSL on the surfaces with open Li channels (Figure 4c and Figure S8). The outer edges show a thin RSL with reduced Ni and little TM inhomogeneity as shown in the EELS and EDX maps in Figure 5c,e. Overall, despite experiencing 300 cycles, the particle is well preserved. Therefore, some of the primary particles are mechanically unaffected by long cycling. However, crystals heavily affected by cycling can also be found in the PCC300 sample, as demonstrated in Figure 5b,d,f. Intragranular pores (red arrow in Figure 5b), intragranular cracks (blue arrows), and steplike features (yellow arrows) as well as regions of strain can be identified. EELS maps in Figure 5d show Ni reduction at the outer surfaces of the crystal, as well as some Ni reduction (to a smaller degree) within the crystal, which could be a sign of severe bulk degradation. Interestingly, compared to all previous EELS maps, in this particle Ni reduction coincides with Mn and Co reduction, which is in line with the results of our previous work,^7^ where more severe aging led to more significant Co and Mn reduction. Additionally, a high degree of TM inhomogeneity, reaching around 70 at. % Mn content, is measured (Figure 5f). There are two distinct regions of material close to NMC811 (upper left of the mapped area) and NMC433 (lower right; the division is highlighted with a dashed curve in Figure 5f) that are separated by a region of intragranular cracking, further supporting the hypothesis of TM inhomogeneity leading to strain and intragranular cracking. Steplike features on the surface of this crystal are highlighted with yellow arrows in Figure 5b (all parts of this primary particle share the same crystallographic orientation). Such steplike features could be indicative of lattice plane gliding along the (003) planes as previously described in the literature.^50^ Briefly, it is suggested that this gliding might be caused by a combination of imperfect formation and sealing of intragranular cracks, forces exerted by neighboring particles, and lower shear yield stress in the charged state can lead to displacement of slabs of NMC811 crystals. Examples of similar, steplike microstructure in *discharged* samples after cycling are shown in Figure 6, highlighting that intragranular cracks, while not causing major degradation early in battery cycle life may, over time, lead to deterioration of the cathode material through lattice plane gliding (which increases the surface area exposed to electrolyte) and inner surface reduction of “open” cracks as discussed previously.

### 2.4 Origins of Intragranular Cracking

Overall, there is significant inhomogeneity in the degree of defectiveness (intragranular cracking, reduction, and reconstruction of inner surfaces of intragranular cracks, and TM inhomogeneity) of NMC crystals, which has not been previously discussed in the literature. It is evident within electrodes (Figure 5), within secondary particles (Figure 4) and even within a single, primary particle (Figures 4, 5b). In both PCCF and PCC300, there are examples of well-“preserved” primary particles, with TM reduction mostly constrained to the outer surfaces and no severe intragranular cracking. However, even after just formation (and after 300 cycles) some primary particles exhibit considerable intragranular cracking (Figure 4a), TM reduction within the primary crystals (Figures 4c,d and 5d), and TM inhomogeneity (Figures 4e,f and 5f). The obvious question comes to mind: how do these primary particles differ that they change in such different ways upon formation and cycling?

**Figure 7:**
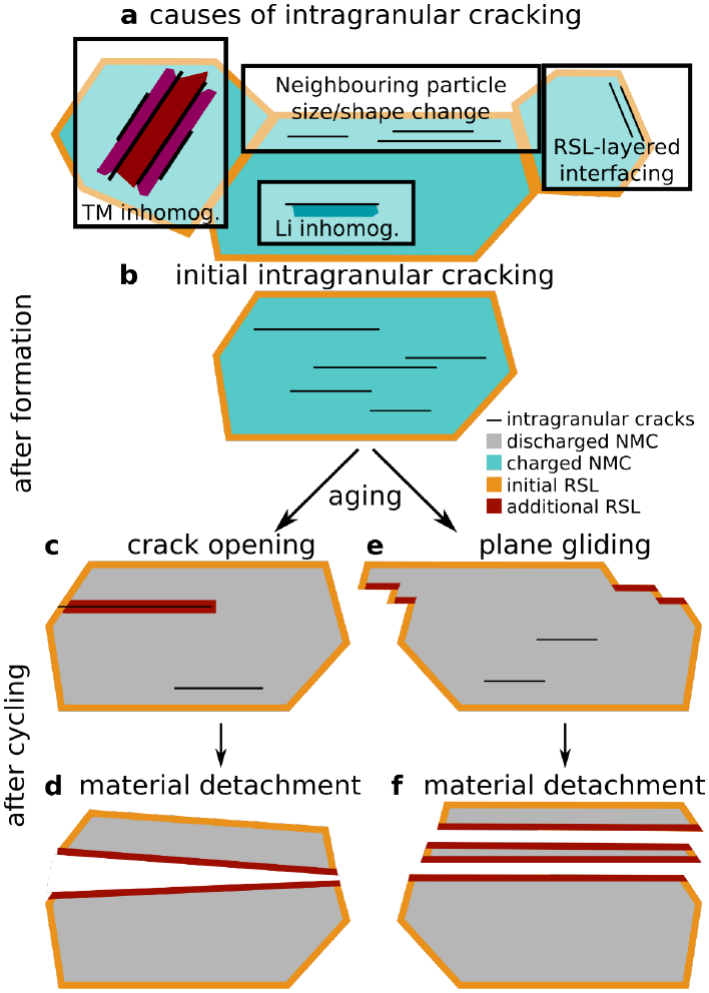
Schematic representation of the evolution mechanisms of intragranular cracks. (a) Charged NMC single crystal viewed along the (003) planes showing initial intragranular cracking and an initial RSL layer on the outer surface (orange). (b–e) Discharged NMC crystals showing detrimental effects of intragranular cracking after aging. (c,d) Effects of crack reaching the outer surface and flooding with the electrolyte. This leads to the formation of additional RSL (dark red) and eventually detachment of a segment of the original crystal. (e,f) Effects of plane gliding that also leads to detachment of sections of the original crystal, and formation of additional RSL.

Our results suggest that intragranular cracking *does not* have a single cause. Rather, we propose that intragranular cracking is generally caused by inhomogeneous stresses within an NMC crystal that can be a result of one or more of the following (as also summarized in Figure 7a):

1 Expansion/contraction of neighboring primary particles (mostly relevant to polycrystalline NMCs).
2 TM inhomogeneity leading to inhomogeneous expansion and contraction within the crystal upon SOC change.
3 Interfacing of the rock-salt and layered phases that have different expansion/contraction behaviors with respect to SOC change.
4. Inhomogeneous Li distribution, leading to different effective SOC and different levels of expansion and contraction (not probed in this work but demonstrated in the literature).^38,51−53^

All of the above sources of stress are more significant with a higher SOC, which can lead to the formation of intragranular cracks in the charged state already after formation. This is exacerbated by the fact that an NMC exhibits lower mechanical strength (tensile/compressive strength and Young’s modulus)^54^ at a high SOC. This SOC dependence is key for intragranular cracking: (i) the intragranular cracks are not observed in calendered, noncycled samples, so application of a “global” stress at low SOC is not enough to cause intragranular cracking (due to higher mechanical strength at low SOC or dissipation of the forces present during calendering by intergranular cracking); (ii) after the SOC-dependent sources of local stress are removed during discharge, the intragranular cracks seal. Importantly, as the sources of local stress are intrinsically inhomogeneous, intragranular cracking as a degradation mechanism is also highly inhomogeneous and is determined by the local environment of every crystal and within each crystal. Lastly, while calendering alone is not sufficient to cause intragranular cracking, it may affect it upon charging as it would increase the interactions between neighboring particles by packing them more tightly (see Figure 2 and Note S1).

### 2.5 Implications for Cathode Performance

Intragranular cracks can negatively impact the battery performance in two main ways, which are schematically shown in Figure 7. First, if an intragranular crack is not perfectly sealed upon discharge and reforms upon subsequent charge, over the course of prolonged cycling it can propagate and reach the surface of a primary crystal leading to flooding with electrolyte and surface reduction (Figure 7c) or to separation of parts of the material (Figure 7d). Second, the repeated formation and sealing of the intragranular cracks can lead to gliding of (003) planes^50^ and potentially even defragmentation of primary particles in a form of thin flakes upon longer cycling (Figure 7e,f). Both of these processes would lead to loss of electrical contact between particles, and to an increase in the surface area available for side reactions and oxygen loss causing additional RSL formation (loss of active material, dark red in Figure 7c–f) as well as electrolyte degradation through oxidation.^8−10^ We also suspect that the reversible formation of intragranular cracks may be connected to previously described degradation mechanism of a “fatigued” phase^38^ (see Note S2). Our findings indicate that significant inter- and intragranular cracking of NMC secondary and primary particles, respectively, happen very early in cycle life (after formation or before cycling in the case of intergranular cracking), and yet, the cracking does not immediately give rise to significant capacity loss or impedance rise. Our results highlight that contrary to previous literature, intragranular cracking appears very early in cycle life and under much milder conditions than typically studied (low upper cutoff voltage, three slow cycles, not elevated temperature), suggesting that initially, intragranular cracks are not a sign or a significant cause of performance degradation. Moreover, plane gliding and delamination of NMC crystals are not widespread after prolonged cycling (Figure S2). Macroscopically, both gliding and crack propagation would manifest as charge-transfer impedance rise and capacity fade, similar to other proposed NMC degradation mechanisms such as RSL evolution or intergranular cracking. Therefore, judging the relative importance of intragranular cracks is very difficult and requires further studies. We also suggest that intragranular cracking may initially be a way of accommodating the stresses associated with drastic and potentially inhomogeneous and anisotropic lattice size change during (de)lithiation and not just a sign of degradation.^36,40^ Nevertheless, it is possible that over prolonged cycling, the repeated formation and sealing of intragranular cracks could lead to exposure of fresh surfaces to electrolyte (see Figure 7) and deterioration of the performance of the cathode, as described above. Strain engineering of NMC cathode materials as highlighted by recent literature significantly slows down the degradation of performance, which may suggest that it leads to a reduction of severity of inter- and intragranular cracking during cycling.^48,55−57^ Moreover, our results reveal previously unexpected heterogeneity in the TM distribution in this commercial material. Improving the homogeneity of the NMC811 particles at the primary and secondary levels during synthesis would also decrease the local strain and intragranular cracking and potentially lead to better performance retention.

## 3 Conclusions

This in-depth electron microscopy study of a comprehensive sample set reveals that intragranular cracks are generally formed in the charged state (already after formation) due to a combination of local stress (often exacerbated in the charged state) and lower mechanical strength and that the cracks largely seal upon discharge. Different intragranular cracks (even within the same crystal) can have different sources of local stress, and the origins of the local stresses have been identified. Our results highlight that neither inter- nor intragranular cracking is immediately detrimental to the performance of the battery, as samples after three formation cycles already exhibit significant inter- and intragranular cracking in the charged state. Intragranular crack propagation and plane gliding may be manifested with prolonged cycling. Open cracks, crack propagation, and plane gliding can lead to increased surface area exposed to electrolyte causing additional oxygen loss, which in turn causes electrolyte degradation, active material loss through surface layer reduction, which combined with loss of electrical contact results (macroscopically) in capacity fade and impedance rise. However, quantification of the relative importance of intragranular cracking, with respect to other degradation mechanisms, is challenging and requires further studies. In summary, this work provides advanced insights into the nature and origins of intragranular cracks, which lead to a deeper understanding of the processes involved and will be crucial in guiding future strategies to mitigate degradation of high-energy NMC-based batteries.

## 4 Methods

### 4.1 Materials and Electrochemistry

Calendered PC NMC811 and graphite electrodes were prepared in the Cell Analysis, Modeling, and Prototyping (CAMP) facility at the Argonne National Laboratory. The cathodes were cast onto Al foil using 90 wt % NMC811 (Targray), 5 wt % polyvinylidene difluoride binder (PVDF; Solvay 5130), and 5 wt % conductive carbon additive (Timcal C45) suspended in *N*-methyl-2-pyrrolidone (NMP) to create a slurry. The anodes consisted of 91.83 wt % artificial graphite (Hitachi MagE3), 2 wt % conductive carbon (Timcal C45), 6 wt % PVDF binder (Kureha 9300), and 0.17 wt % oxalic acid cast onto 10-μm-thick copper foil using NMP as the solvent. After drying, the electrodes were calendered using a heated (80 °C) two-roller hydraulic-driven roll press (A-PRO Co.) to 30% porosity. Additionally, single-crystal NMC811 (LiFun) and polycrystalline NMC811 (Targray) powders were mixed in a centrifugal mixer (Thinky) with conductive carbon (C45, Timcal) and binder (polyvinylidene difluoride, MTI) in the same wt % ratio as above to form PCNF and SCNF cathodes accordingly. The powders were dispersed in NMP to form a viscous slurry, which was then cast on Al foil. The electrodes were dried in a vacuum oven at 120 °C. Disks 14 mm in diameter were cut, weighed, dried, and transferred directly to an Ar-filled glovebox. The average mass loading for PC NMC was 8.22 or 8.90 mg/cm^2^ for calendered and noncalendered electrodes, respectively. SC NMC electrodes were noncalendered and had an areal loading of 8.18 mg/cm^2^. These mass loadings correspond to 1.52, 1.65, and 1.51 mAh/cm^2^ for PC calendered, PC noncalendered, and SC noncalendered electrodes, respectively (assuming 185 mAh/g_NMC811_). Full cells were assembled by using a glass fiber separator (16 mm in diameter, GF/A, Whitman) and an additional Celgard separator (16 mm in diameter) placed on the cathode to ensure easy separation of the electrode after cycling. Graphite electrodes (15 mm diameter, dried in a vacuum oven at 120 °C, with loading of 5.83 mg_Gr_ cm^–2^ corresponding to ∼1.92 mA h cm^–2^ based on 330 mA h g_Gr_^–1^) and 80 μL of LP57 (1 M LiPF_6_ in ethylene carbonate (EC):ethyl methyl carbonate (EMC) 3:7 v/v) were used as the anode and electrolyte, respectively.

Formation in full cells was common for all cycled specimens (PCCF, PCNF, SCNF, PCC300): three formation cycles (constant current–constant voltage charge – CCCV, with C/40 current cutoff and constant current – CC, discharge) of NMC811/graphite full cells were performed between 2.5 and 4.2 V at C/20 rate (assuming a practical capacity of 185 mAh/g). To assess the effect of longer cycling, after formation some of the full cells (PCC300) were subjected to 300 cycles at C/2 rate between 2.5 and 4.2 V (CCCV charge with C/20 current cutoff and CC discharge). These voltage ranges were chosen to stay within realistic cutoff conditions without exacerbating early degradation of NMC by going to very high upper cutoff voltage. After the full cell cycling (formation or formation +300 cycles), the cells were decrimped, the cathode was extracted and immediately (without washing) made into half cells with Li metal as the counter electrode and a fresh Celgard separator soaked in 40 μL of fresh LP57 electrolyte. The half cells were then charged to 4.3 V versus Li at C/20 and held at the UCP for 10 h to equilibrate. This step was crucial to ensure that the resulting SOC is the same for all samples regardless of their cycling history. If the cells were charged to a fixed voltage in the original full cells (vs graphite), the NMC potential (and therefore, SOC) would not be accurately known and may vary between samples based on cycling history.

### 4.2 Sample Preparation

After cycling and charging to 4.3 V vs Li in half-cell configuration, the coin cells were decrimped in an Ar-filled glovebox (<0.5 ppm of O_2_ and H_2_O). The cathode was harvested, washed with dimethyl carbonate (anhydrous, Sigma-Aldrich), dried in dynamic vacuum of a glovebox antechamber for 30 min, and left to dry overnight inside of the glovebox. Electrodes prepared in this way were cut into smaller pieces for different experiments. For STEM-EELS and HR-STEM experiments, the dry cathodes were transferred into a FIB-SEM (FEI Helios NanoLab) in an Ar-filled sealed bag. There, standard TEM lamellae were cut out of the cathode. Secondary particles that were not obviously damaged by calendering (based on top view SEM imaging) were chosen. This is to avoid excessive intergranular cracking that could cause mechanical instability of the resulting lamellae. The lamellae were attached to Cu TEM half-grids, thinned to <100 nm and transported to a glovebox. The samples were double sealed (sealed bag within a sealed bag) in Ar for transportation to Trondheim, Norway, where the STEM-EELS and HR-STEM experiments were performed. The total air exposure time was 10 min for FIB lamellae preparation and 1 min for transfer into the TEM. For FIB-SEM tomography, the samples were simply sealed in Ar-filled bags and transferred to the microscope (air exposure time: 1 min).

### 4.3 Electron Microscopy

For the FIB-SEM tomography experiments, a Zeiss CrossBeam 540 dual-beam microscope was used. A 1-μm-thick Pt-based protective layer was deposited on the top surfaces of the cathodes. Then, alignment marks were milled in the Pt layer and covered with a 1 μm carbon layer. Initial FIB rough milling to prepare a 20 × 30 × 30 μm volume was made using a 30 kV Ga ion beam at 30 nA beam current. Then, the milling of tomographic slices (each slice about 50 nm in nominal thickness) was done using a 30 kV 1.5 nA Ga ion beam. After each slice, a backscattered electron image was acquired using 2.6 kV accelerating voltage and 12 nA beam current at about 25 nm pixel size.

A JEOL ARM 200FC transmission electron microscope operated at 200 kV accelerating voltage was used for high-resolution imaging and electron energy loss spectroscopy in scanning mode experiments. The microscope is equipped with a cold field-emission gun. The microscope is probe- and image-corrected (although in these experiments only the probe correction is relevant). The imaged and mapped crystals were first aligned so that the (003) planes of the NMC are parallel to the electron beam. The spectra were collected by using a Gatan Quantum ER spectrometer. Dual-EELS mode was used at 0.25 eV/channel dispersion to collect the low- (including the zero-loss peak, ZLP) and core-loss spectra simultaneously at each probe position, forming a spectrum image. Pixel sizes of about 2 nm and 200 ms dwell time were typically used. The energy resolution was determined as the full width at half-maximum of the ZLP and was about 1 eV. EELS collection semiangle of 66.86 mrad was used. Energy-dispersive X-ray spectrum images were also acquired simultaneously with the EEL spectrum images using a Centurio SDD EDX detector with a solid angle of 0.98 sr. Imaging was performed using two annular dark-field detectors: (1) high-angle annular dark field (HAADF) with a collection angle between 118.50 and 470 mrad and low-angle annular dark field (LAADF) with the outer collection angle limited by the HAADF detector: 67.42–118.50 mrad. HAADF images provide atomic number–thickness contrast, while LAADF contains some additional contrast from strain. These parameters were chosen to map the strain inhomogeneities in the probed samples by comparing the HAADF and LAADF images (Figures S4 and S5). FEI Tecnai F20 microscope operated at 200 kV and equipped with a Gatan OneView 4k camera was used for bright-field TEM imaging.

### 4.4 Data Processing

EEL spectrum images were preprocessed as described in our previous work.^7^ Briefly, the spectrum images (SI) were processed using HyperSpy^58^ (a Python library for multidimensional data analysis). First, any unexpected spikes (cosmic rays, stray X-rays hitting the detector) in the data were removed, followed by the zero-loss peak alignment. The zero-loss peak position for each pixel of the SI was used to shift both low- and core-loss spectra, so the center of the zero-loss peak was at 0 eV energy loss. The region of the core-loss spectrum before the onset of the oxygen K-edge was used to fit and subtract a power law background. The TM L edges were fitted at each pixel with (i) a power law background, (ii) two Hartree–Slater generalized-oscillator-strength-based ionization edges, (iii) two Gaussian peaks (for the L_3_ and L_2_ white line peaks), and (iv) their convolution with the low-loss peak to account for the sample thickness. Maps of the TM L_3_ Gaussian peak position were produced. These maps provide information about the oxidation states of the TMs, as the TM L_3_/L_2_ edges correspond to transitions between 2p and unfilled 3d orbitals, which manifest as two peaks (L_3_ and L_2_) due to spin–orbit splitting. In this work, we focus on the chemical shifts of the L_3_ peak, as other methods (e.g., peak intensity ratio)^59,60^ require high signal-to-noise ratios, which need higher electron doses, potentially leading to sample damage. TM L_3_ peak shifts toward lower electron energy losses correspond to a reduction of that TM.^59,60^ The Mn and Co EEL spectra were binned by a factor of 2 in both spatial directions to improve the SNR (they are 4 times less abundant than Ni in NMC811 and therefore the intensity of their edges is significantly lower). The EDX spectrum images were also analyzed in HyperSpy by numerically integrating the TM X-ray lines. The integrated X-ray line intensities were then used as inputs to the Cliff–Lorimer method by using k-factors provided by the detector manufacturer.

## Data Availability

The data that support the findings of this study are available from the corresponding author upon reasonable request.

## Supporting Information Available

Additional FIB-SEM and (S)TEM images of SC and PC samples in the discharged state, further discussion about intergranular cracks at different SoC, additional LAADF-STEM micrographs highlighting strain in the samples, STEM-EDX mapping of pristine PC NMC811, STEM-HAADF micrographs showing the thickness of the surface layer at different crystal facets, and discussion about the possible connection of the NMC811 fatigue and intragranular cracking (PDF)
