# Supplementary material for: Origins and Importance of Intragranular Cracking in Layered Lithium Transition Metal Oxide Cathodes
Source: ACS Appl Energy Mater. 2024 Apr 25;7(9):3945–56. doi: 10.1021/acsaem.4c00279 (PMC11094680; doi:10.1021/acsaem.4c00279)
Supplement: Supplementary file 1 — ae4c00279_si_001.pdf [file ae4c00279_si_001.pdf]

Supporting information

Origins and importance of intragranular cracking in layered lithium transition metal oxide cathodes

Jędrzej K. Morzy[1,2,3], Wesley M. Dose[2,3,4], Per Erik Vullum[5,6], May Ching Lai[1,3], Amoghavarsha Mahadevegowda[1,3], Michael F. L. De Volder[2,3], Caterina Ducati[1,3,*]

[1] Department of Materials Science and Metallurgy, University of Cambridge, 27 Charles Babbage Road, CB3 0FS Cambridge, United Kingdom

[2] Institute for Manufacturing, Department of Engineering, University of Cambridge, 17 Charles Babbage Road, CB3 0FS Cambridge, United Kingdom

[3] Faraday Institution, Quad One, Harwell Science and Innovation Campus, Didcot, United Kingdom

[4] Current address: School of Chemistry, University of New South Wales, Sydney 2052, Australia

[5] Department of Physics, Norwegian University of Science and Technology, Høgskoleringen 1, 7034 Trondheim, Norway

[6] Sintef Industry, Høgskoleringen 5, 7034 Trondheim, Norway

*corresponding author, email: cd251@cam.ac.uk

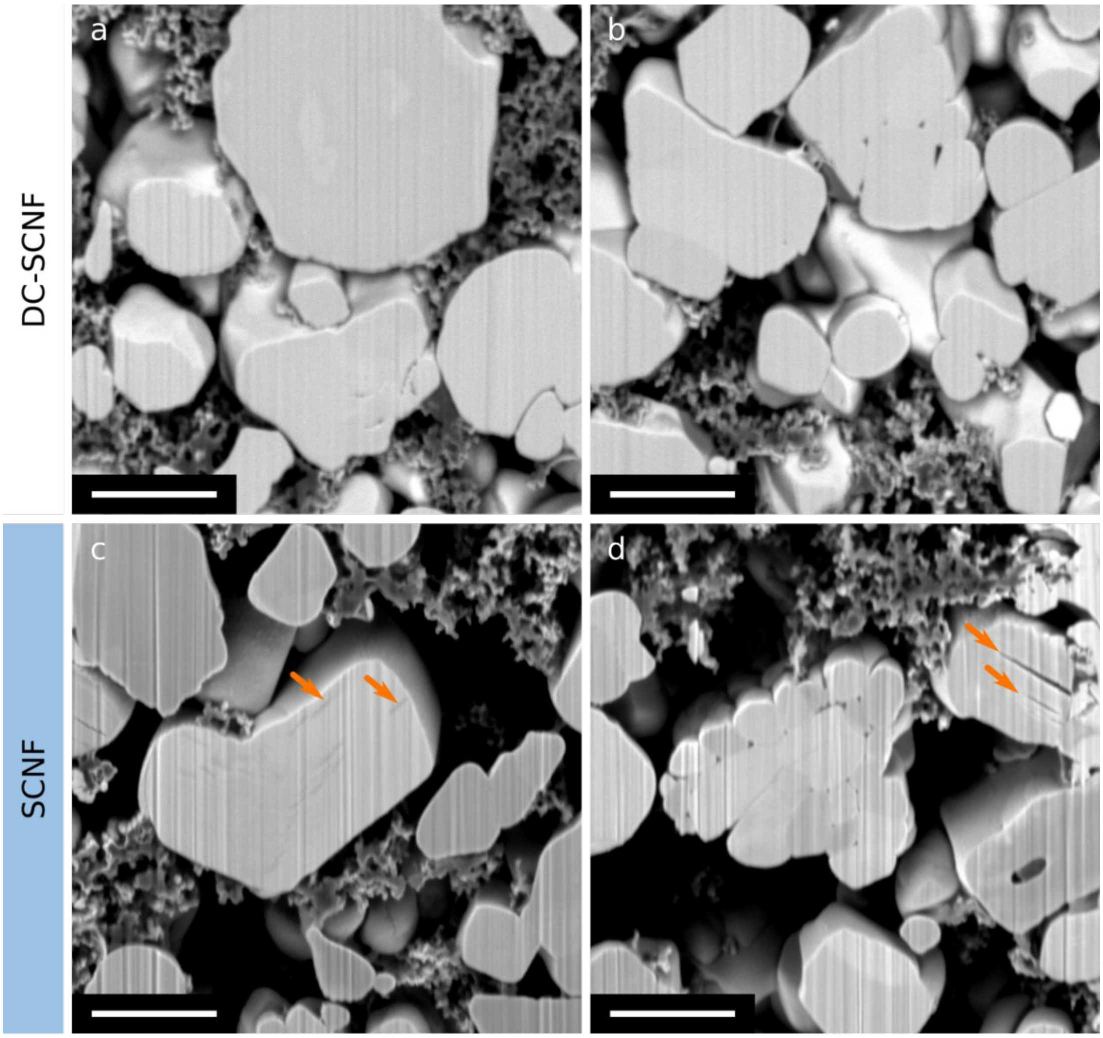

Fig. S1 Cross-sectional SEM images of single crystal, non calendered NMC cathodes after formation sample in discharged (DC-SCNF, a-b) and charged (SCNF, c-d) state. (a-b) backscattered electron detector, (c-d) Everhart-Thornley detector (secondary and backscattered electrons). Intragranular cracks are highlighted in (c-d) (orange arrows). Scale bars are 2 µm. Vertical lines are curtaining artefact from the FIB milling process.

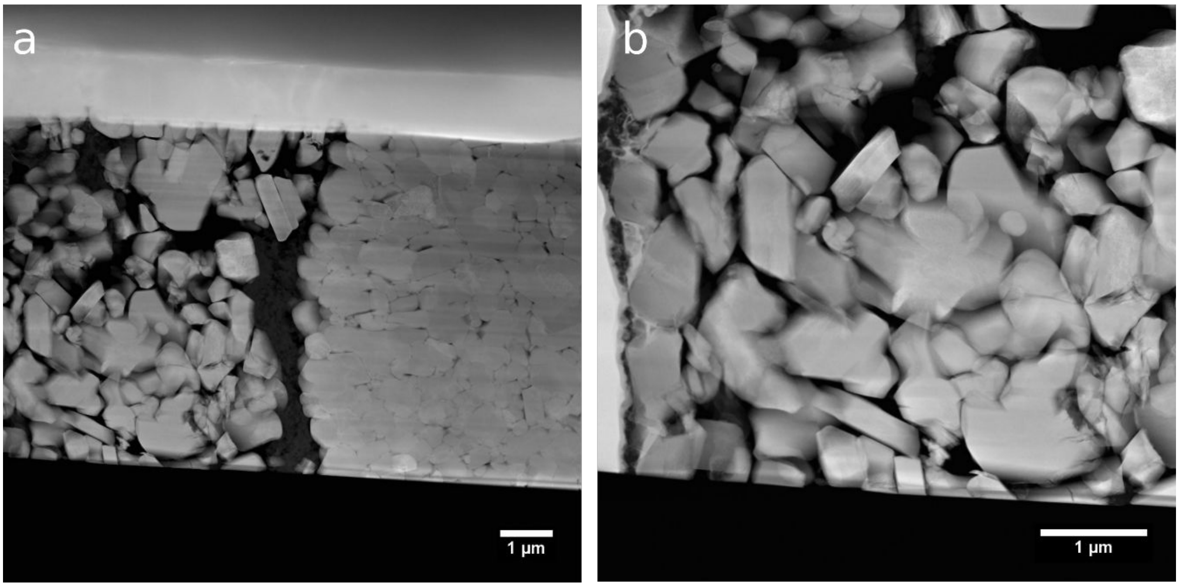

Fig. S2. (a) low magnification and (b) medium magnification HAADF-STEM images of a discharged NMC811 lamella after 100 C/3 cycles vs graphite between 2.5 and 4.2 V. Intragranular cracks in the discharged state are not commonly observed.

Supporting Note S1 – intergranular cracking in non-calendered electrodes

Fig. 2 c shows that non-calendered secondary particles of NMCs are more severely affected by intergranular cracking in the charged state. Their grain boundaries are 'wider' (these gaps are intergranular cracks present throughout the secondary particle)and the secondary particles are less densely packed compared to their calendered counterparts (Fig. 2 b). In the charged state, the lattice of NMC811 has undergone significant shrinkage in all directions. The *a* cell parameter shrinks monotonically by about 2.2%, while *c* unit cell parameter initially expands by 1.7% (until NMC is charged to ~70% SOC) and then shrinks by about 3.4% of its initial size. Unit cell volume shrinks by 5.5% with respect to the discharged state size.[1] Such anisotropic volume change of all particles may provide enough stress at the interfaces of primary particles to result in intergranular cracking.[2–4] The addition of calendering could contribute to better cohesion of secondary particles with each other and the carbon-binder matrix and therefore largely prevent this degree of intergranular cracking present in charged

state after formation.[5,6] This is somewhat counterintuitive and could explain the worse performance of non-calendered electrodes (higher capacity fade, polarisation and impedance rise during cycling between 2.5-4.3 V vs graphite, in constant current – constant voltage mode at C/2 rate)[5]: calendering introduces intergranular cracking and secondary particle pulverisation prior to cycling, which can increase the NMC811 specific surface area, and lead to loss of electrical contact in some extreme cases, but also to improvement of rate capability by shortening the average path Li ions need to take through solid state diffusion to get into all parts of the active material.[1,5,7] In turn, not calendering the electrode might not immediately introduce intergranular cracking in the discharged state prior to cycling, but on the other hand the intergranular cracking observed after formation in the charged state is much more severe for non-calendered electrodes and is more likely to develop into irreversible cracking in later cycles due to lower cohesion within the secondary particles.[5,6] Moreover, this early life severe intergranular cracking (or opening of grain boundaries) would lead to increased surface area exposed to electrolyte and more side reactions. Lastly, this closer packing of crystals in secondary particles in the charged state for calendered electrodes could impact intragranular cracking as it would increase the interactions of anisotropic expansion and contraction of the neighbouring crystals.

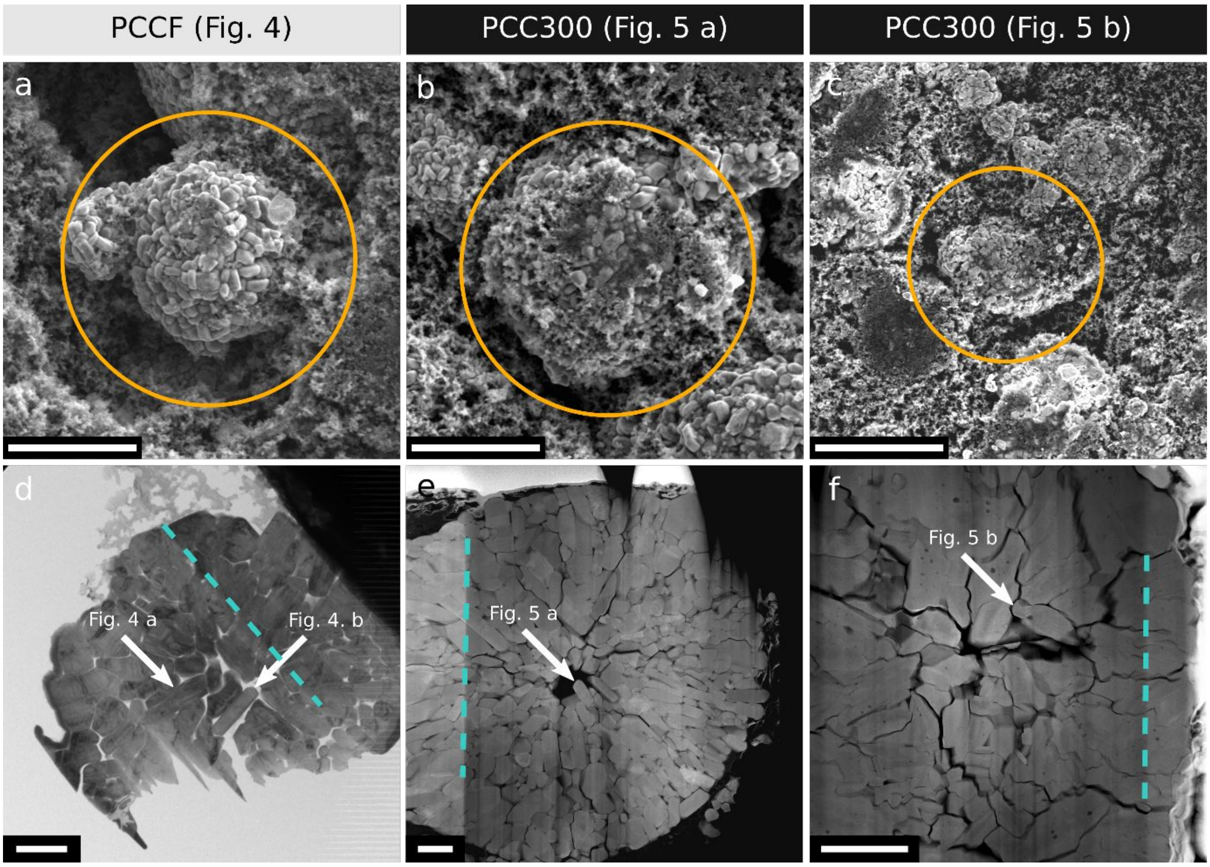

Fig. S3 (a-c) Top-down SEM images (secondary electron detector), (d) bright-field TEM, and HAADF-STEM (e-f) images of NMC811 secondary particles chosen for HR-STEM and STEM-EELS-EDX analysis (highlighted by orange circles in (a-c). In (d-f) white arrows point towards particular primary particles analysed in this work. Teal dashed lines show the direction of the FIB milling, which is parallel to the curtaining effect. Scale bar in top row (a-c) is 5 µm and in bottom row (d-f) 2 µm. Secondary particles not protruding out of the electrode were chosen for FIB lamellae preparation as to avoid secondary particles that were pulverised by calendering.

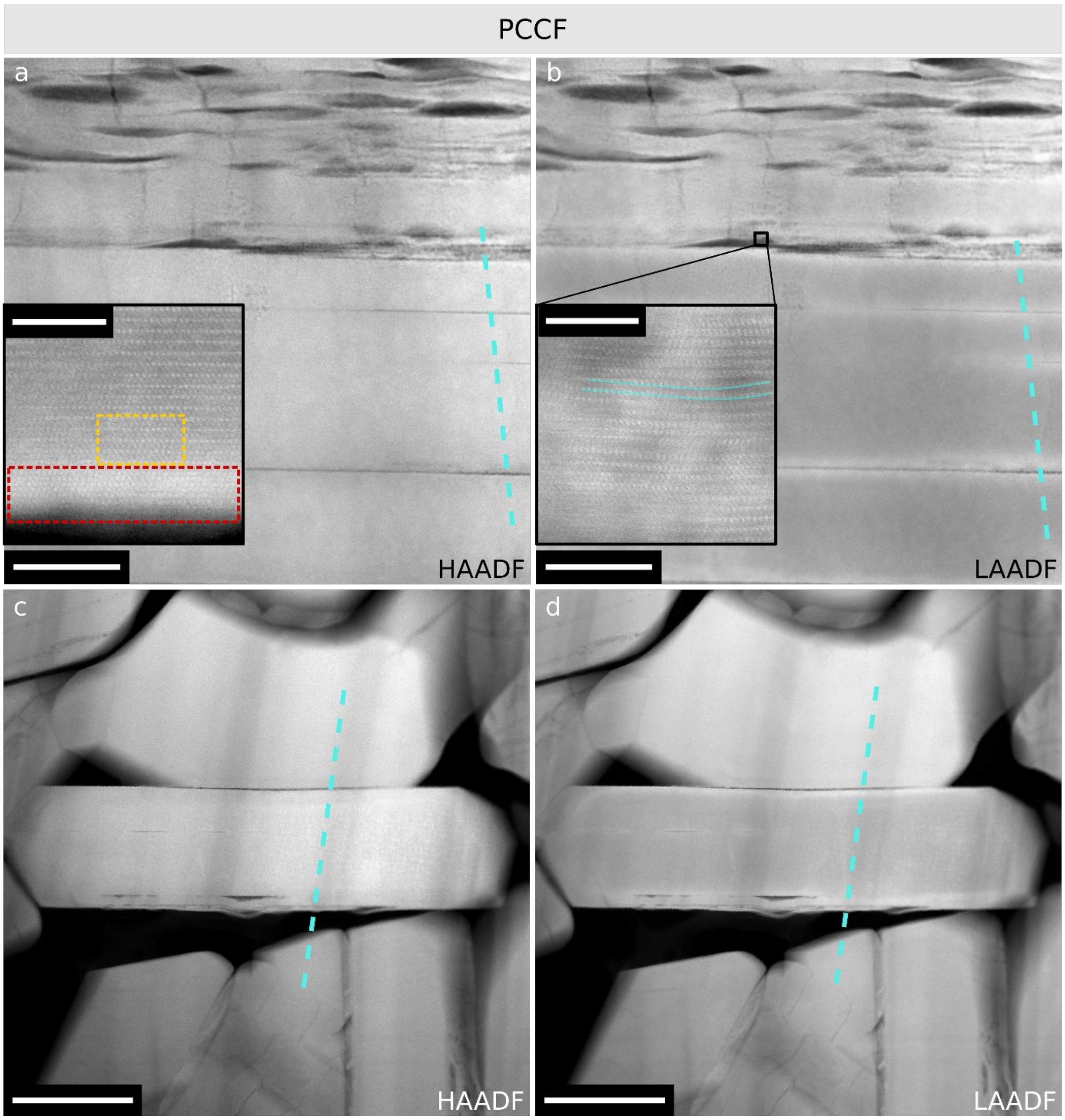

Fig. S4. LAADF and HAADF STEM images of the same areas in PCCF sample (see Fig. 4). The (003) planes are oriented close to horizontally in both images. Inset in (a) is from the bottom edge of this crystal (not shown in a). Inset in (b) highlights lattice distortions around intragranular cracks. Scale bar in (a-b) 50 nm, (c-d) 200 nm, insets in (a,b) 5 nm. Teal dashed lines highlight the direction of curtaining, which is an artefact of the lamellae preparation process (FIB milling) and introduces differences in contrast in bands along that direction.

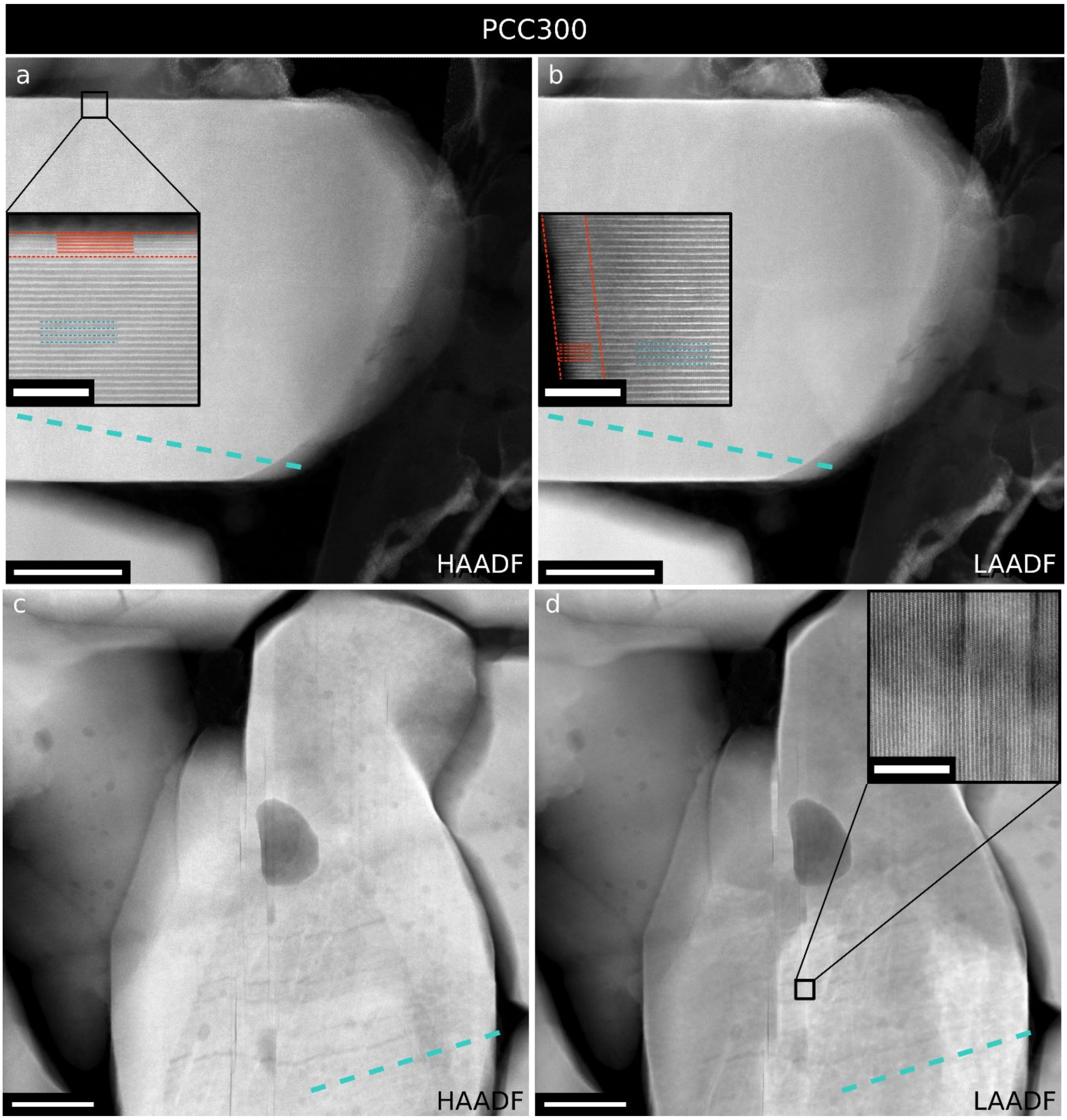

Fig. S5. LAADF and HAADF STEM images of the same areas in PCC300 sample (see Fig. 5). The (003) planes are oriented close to horizontally in (a-b) and vertically in (c-d). Insets in (a-b) demonstrate high quality layered structure in this crystal with thin RSL at two types of surfaces: parallel to (003) planes (a) and across the (003) planes of NMC (b). Inset in (d) highlights the atomic structure of intragranular cracks and a lack of RSL on their surfaces. Scale bar in (a-d) 50 nm, insets in (a-b): 5 nm. Teal dashed lines highlight the direction of curtaining, which is an artefact of the lamellae preparation process (FIB milling) and introduces differences in contrast in bands along that direction.

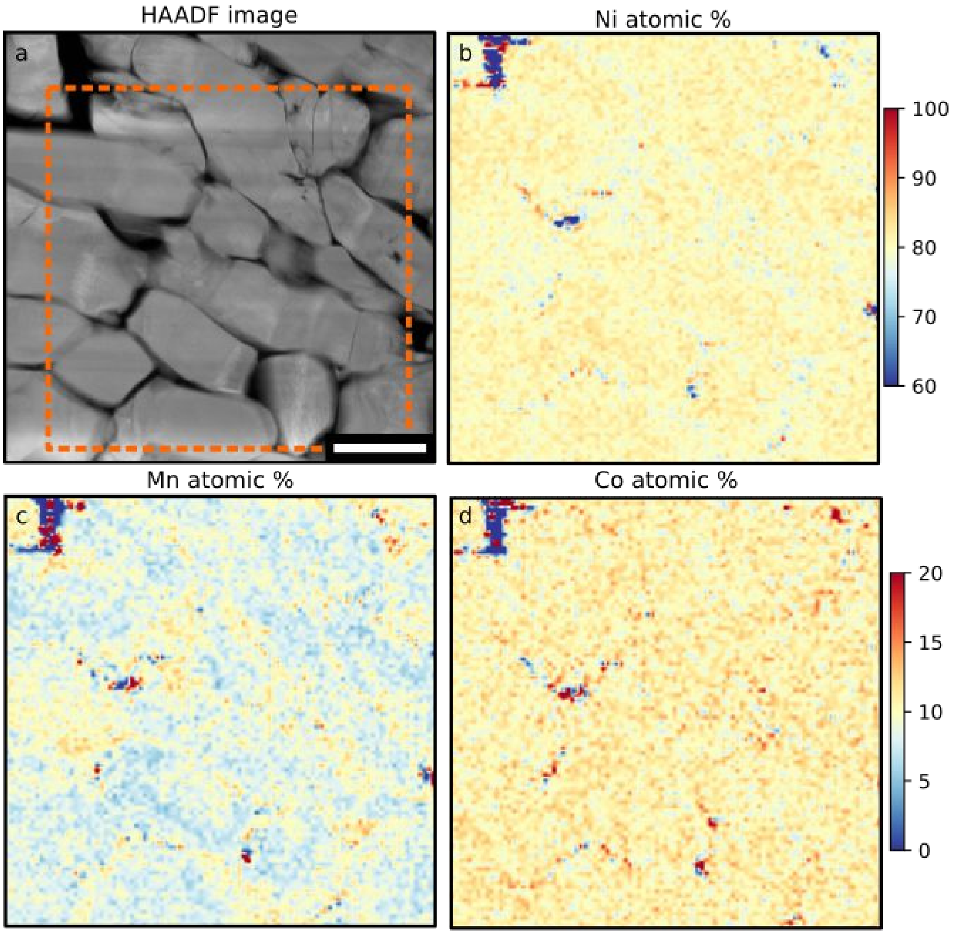

Fig. S6. STEM-EDX mapping of a PCC NMC811 cathode. (a) HAADF image of the investigated region (FIB lamella of a secondary particle). (b-d) TM atomic concentration maps from the region highlighted with a red square in (a). Note the colour scale change between (b) Ni and (c-d) Mn and Co. The scale bar in (a) is 0.5 μm.

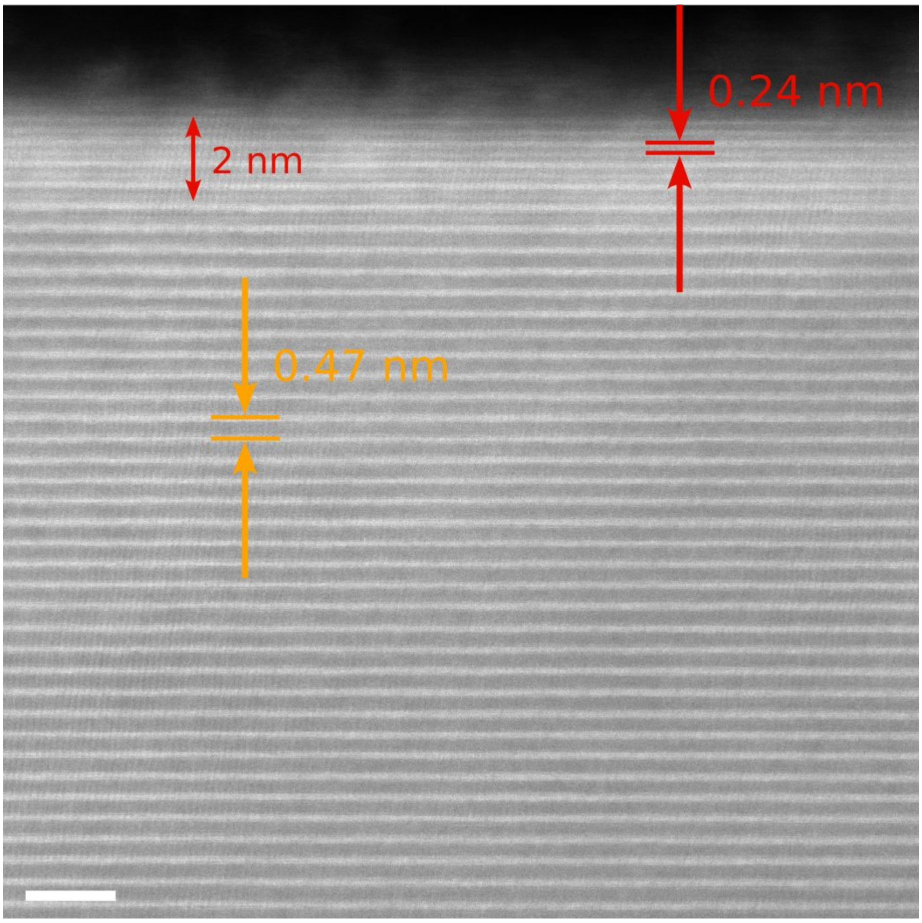

Fig. S7. HAADF-STEM image of the 'top' edge of the same PCC300 particle as shown in Fig. 5 a. The image highlights the different lattice spacing of the RSL and layered structures (red and yellow respectively). The thickness of the RSL on the (003) terminated facet is approximately 2 nm. Scale bar – 2 nm. A layered structure is maintained throughout the crystal (except the thin surface layers) despite 300 cycles after formation.

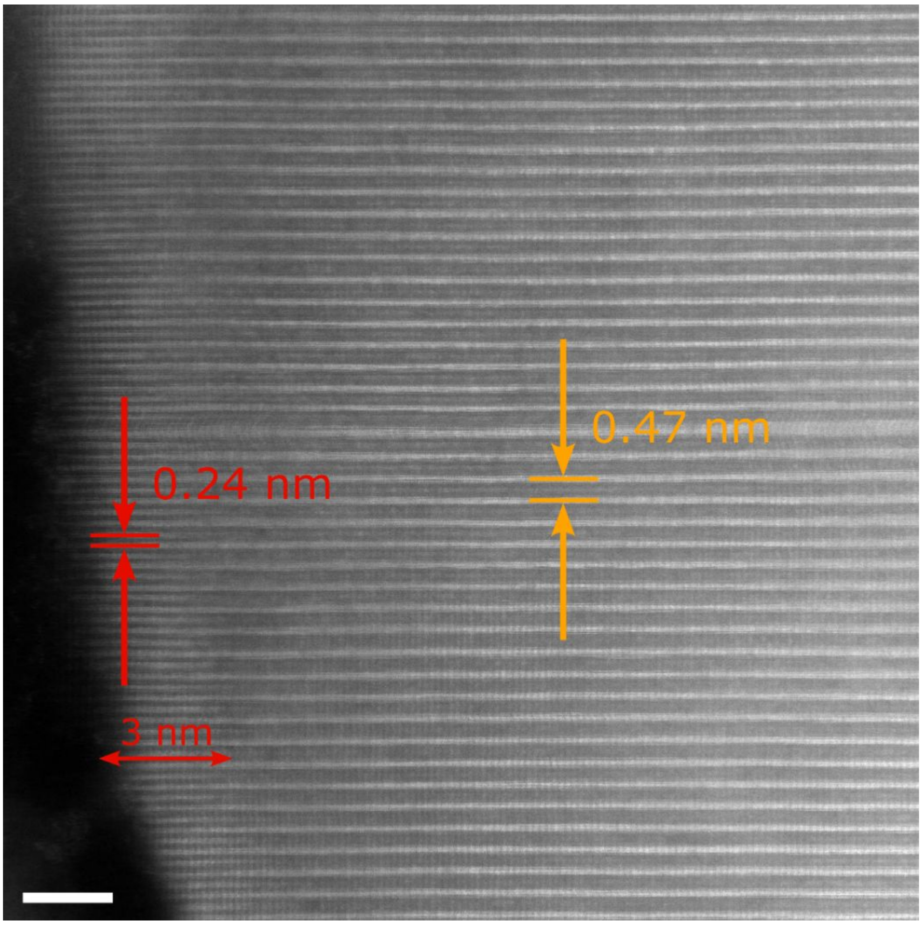

Fig. S8. HAADF-STEM image of the 'left-hand' edge of the same PCC300 particle as shown in Fig. 5 a. The image highlights the different lattice spacing of the RSL and layered structures (red and yellow respectively). The thickness of the RSL along the open channels is approximately 3 nm. Scale bar – 2 nm. A layered structure is maintained throughout the crystal (except the thin surface layers) despite 300 cycles after formation.

Supporting Note 2 – fatigued phase and intragranular cracks

It is possible that one of the signs of long-term degradation caused by intragranular cracking is connected to a finding by Xu at al.,[8] who reported, based on operando XRD measurements, that upon charging of NMC811/graphite cells after aging (348 cycles, 2.5-4.2 V) a fraction of NMC811 (10-20%, which increases with number of cycles) does not reach high SOC that would be expected based on the voltage of the cell. This fraction is called the

'fatigued' phase and in their work is attributed to pinning of the layered lattice by the thin RSL layer at the surfaces of NMC811. Such pinning is proposed to prevent the layered structure lattice collapse and Li extraction at high SOC. However, based on our results, an alternative explanation can be proposed: the 'fatigued' phase is caused by intragranular cracks and segmentation of the primary NMC crystals. As the intragranular cracks appear at high SOC and largely seal upon discharging, it is possible that at high SOC intragranular cracks are large and disruptive enough, to mostly isolate certain parts of the active material. Then, the isolated NMC fragments would only be electrochemically active up to a certain voltage (or SOC), above which they become inactive due to lack of good connection pathway to the current collector. As the intragranular cracks exhibit large reversibility (i.e. forming and sealing on charge and discharge respectively), some of the isolated material would become active again once it is reconnected upon closing of cracks (as the voltage and SOC are lowered again). During aging, the density of intragranular cracking may increase, leading to a larger proportion of the 'fatigued' phase, inactive at high SOC. On the other hand, in a case where the cracks are not fully reversible, parts of the material would become more permanently inactive, leading to a loss of available cathode capacity.

References:

1. Märker, K., Reeves, P. J., Xu, C., Griffith, K. J. & Grey, C. P. Evolution of Structure and Lithium Dynamics in $LiNi_{0.8}Mn_{0.1}Co_{0.1}O_2$ (NMC811) Cathodes during Electrochemical Cycling. *Chemistry of Materials* **31**, 2545–2554 (2019).

2. Oswald, S., Pritzl, D., Wetjen, M. & Gasteiger, H. A. Novel Method for Monitoring the Electrochemical Capacitance by In Situ Impedance Spectroscopy as Indicator for Particle Cracking of Nickel-Rich NCMs: Part II. Effect of Oxygen Release Dependent on Particle Morphology. *J Electrochem Soc* **168**, 120501 (2021).

3. Hu, J. *et al.* Mesoscale-architecture-based crack evolution dictating cycling stability of advanced lithium ion batteries. *Nano Energy* **79**, 105420 (2021).

4. Trevisanello, E., Ruess, R., Conforto, G., Richter, F. H. & Janek, J. Polycrystalline and Single Crystalline NCM Cathode Materials—Quantifying Particle Cracking, Active Surface Area, and Lithium Diffusion. *Adv Energy Mater* **2003400**, 2003400 (2021).

5. Sim, R., Lee, S., Li, W. & Manthiram, A. Influence of Calendering on the Electrochemical Performance of $LiNi_{0.9}Mn_{0.05}Al_{0.05}O_2$ Cathodes in Lithium-Ion Cells. *ACS Appl Mater Interfaces* **13**, 42898–42908 (2021).

6. Zheng, H., Tan, L., Liu, G., Song, X. & Battaglia, V. S. Calendering effects on the physical and electrochemical properties of $Li[Ni_{1/3}Mn_{1/3}Co_{1/3}]O_2$ cathode. *J Power Sources* **208**, 52–57 (2012).

7. Merryweather, A. J., Schnedermann, C., Jacquet, Q., Grey, C. P. & Rao, A. Operando optical tracking of single-particle ion dynamics in batteries. *Nature 2021 594:7864* **594**, 522–528 (2021).

8. Xu, C. *et al.* Bulk fatigue induced by surface reconstruction in layered Ni-rich cathodes for Li-ion batteries. *Nat Mater* **20**, 84–92 (2021).
